# Mechanism of *trans*-translation revealed by *in vitro* studies

**DOI:** 10.3389/fmicb.2014.00065

**Published:** 2014-02-20

**Authors:** Hyouta Himeno, Daisuke Kurita, Akira Muto

**Affiliations:** ^1^Department of Biochemistry and Molecular Biology, Faculty of Agriculture and Life Science, Hirosaki UniversityHirosaki, Japan; ^2^RNA Research Center, Hirosaki UniversityHirosaki, Japan

**Keywords:** *trans*-translation, tmRNA, SmpB, ribosome rescue system, molecular mimicry

## Abstract

tmRNA is a bacterial small RNA having a structure resembling the upper half of tRNA and its 3′ end accepts alanine followed by binding to EF-Tu like tRNA. Instead of lacking a lower half of the cloverleaf structure including the anticodon, tmRNA has a short coding sequence for tag-peptide that serves as a target of cellular proteases. An elaborate coordination of two functions as tRNA and mRNA facilitates an irregular translation termed *trans*-translation: a single polypeptide is synthesized from two mRNA molecules. It allows resumption of translation stalled on a truncated mRNA, producing a chimeric polypeptide comprising the C-terminally truncated polypeptide derived from truncated mRNA and the C-terminal tag-peptide encoded by tmRNA. *Trans*-translation promotes recycling of the stalled ribosomes in the cell, and the resulting C-terminally tagged polypeptide is preferentially degraded by cellular proteases. Biochemical studies using *in vitro trans*-translation systems together with structural studies have unveiled the molecular mechanism of *trans*-translation, during which the upper and lower halves of tRNA are mimicked by the tRNA-like structure of tmRNA and a tmRNA-specific binding protein called SmpB, respectively. They mimic not only the tRNA structure but also its behavior perhaps at every step of the *trans*-translation process in the ribosome. Furthermore, the C-terminal tail of SmpB, which is unstructured in solution, occupies the mRNA path in the ribosome to play a crucial role in *trans*-translation, addressing how tmRNA·SmpB recognizes the ribosome stalled on a truncated mRNA.

## INTRODUCTION

**FIGURE 1:**
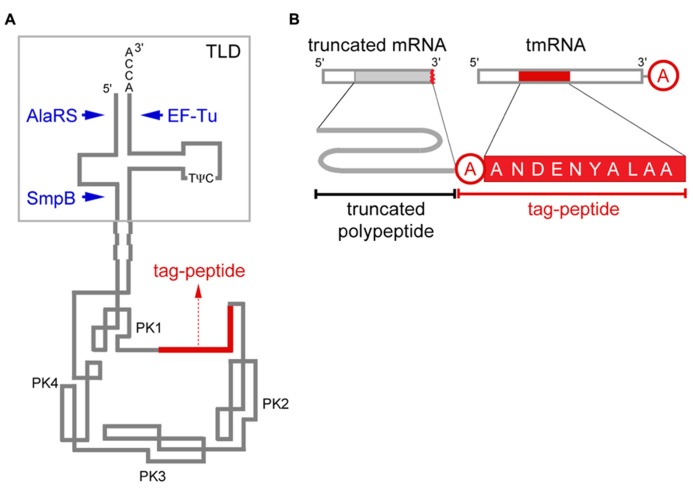
**tmRNA and *trans*-translation. (A)** Secondary structure model of tmRNA. The 3′-end CCA sequence, amino acid acceptor stem, D-arm and T-arm with two typical tRNA-specific modified nucleotides, 5-methyluridine (T) and pseudouridine (ψ; Felden et al., 1998), are present in TLD of *E. coli* tmRNA to which alanyl-tRNA synthetase (AlaRS), EF-Tu and SmpB bind. The tag-encoding sequence (red) is surrounded by four pseudoknot structures (PK1–PK4). **(B)** Schematic representation of *trans*-translation. The tag-peptide sequence varies depending on the bacterial species, and that of *E. coli* is presented. A short peptide encoded by tmRNA is attached to the truncated C-terminus of a polypeptide from truncated mRNA with an alanine residue (circled in red) that is derived from the alanine moiety aminoacylated to tmRNA in between them.

As mRNA acts as a messenger of the genetic information encoded by the genome, while tRNA acts as a tool for decoding, it is reasonable that they are separated molecules. However, both features are equipped with a single small RNA molecule called tmRNA (also known as 10Sa RNA or SsrA). The presence of the tRNA-like secondary structure with several tRNA-specific sequences including the 3′-terminal CCA sequence in tmRNA was first found in 1994 (Komine et al., 1994; Ushida et al., 1994; **Figure 1A**), and thereafter several other structural and functional similarities to tRNA, such as the aminoacylation capacity with alanine (Komine et al., 1994; Ushida et al., 1994), the binding capacity to the ribosome (Ushida et al., 1994; Tadaki et al., 1996), the 5′ processing by RNase P (Komine et al., 1994) and the binding capacity to EF-Tu after aminoacylation (Rudinger-Thirion et al., 1999; Barends et al., 2000, 2001; Hanawa-Suetsugu et al., 2001) and the presence of tRNA-specific base modifications (Felden et al., 1998), have been reported, although the anticodon has never been found in tmRNA, a few hundred nucleotides in length. The function as mRNA has been suggested by an observation that a peptide of 10-amino acid sequence encoded by tmRNA is attached to the truncated C-termini of polypeptides that are exogenously expressed in *Escherichia coli* with an alanine residue of unknown origin in between them (Tu et al., 1995; Keiler et al., 1996; **Figure 1B**). Thus the *trans*-translation model has been proposed: Ala-tmRNA receives the nascent polypeptide from peptidyl-tRNA stalled on a truncated mRNA to continue translation by exchanging the template from truncated mRNA to the tag-encoding region on tmRNA (Keiler et al., 1996). It can address the missing origin of the first alanine residue of the tag-peptide, which is derived from the alanine moiety aminoacylated to tmRNA. This model has been supported by an *in vitro* study showing that the tmRNA-encoded tag-peptide is synthesized using *E. coli* cell extract depending on the presence of poly(U) as a truncated mRNA and on the aminoacylation capacity of tmRNA (Muto et al., 1996; Himeno et al., 1997; Nameki et al., 1999a). The C-terminal four amino acid sequence of the tag-peptide, ALAA, serves as a target for a periplasmic protease, Tsp (Keiler et al., 1996), and cytoplasmic ATP-dependent proteases (AAA+ proteases) including ClpXP, ClpAP, FtsH, and Lon (Gottesman et al., 1998; Herman et al., 1998; Flynn et al., 2001; Choy et al., 2007). In fact, *trans*-translation products, namely, tagged polypeptides, are hardly detectable but become accumulated in cell when the terminal ALAA sequence of the tag-peptide is engineered (Roche and Sauer, 2001; Collier et al., 2002; Fujihara et al., 2002). tmRNA is perhaps absolutely distributed among bacteria and it has also been found in chloroplasts or mitochondria of some eukaryotes (Gueneau de Novoa and Williams, 2004; Jacob et al., 2004), but not in the cytoplasm of eukaryotes or archaebacteria. Taken together, *trans*-translation has been considered as the bacterial system that facilitates recycling of the ribosome stalled on a truncated mRNA lacking a stop codon and to prevent the premature polypeptide from accumulation by adding a tag to its C-terminus for degradation (Keiler et al., 1996; Muto et al., 1998) as well as to promote decay of causative truncated mRNA (Yamamoto et al., 2003). In addition, *trans*-translation has been shown to participate in a wide variety of cellular events (Keiler, 2008), e.g., cell viability (Hutchison et al., 1999; Huang et al., 2000; Akerley et al., 2002; Ramadoss et al., 2013a), stress response (Muto et al., 2000; Ranquet and Gottesman, 2007; Shin and Price, 2007), cell cycle (Keiler and Shapiro, 2003a,b) and regulation of gene expression (Abo et al., 2000; Abe et al., 2008; Ujiie et al., 2009; Chadani et al., 2011a; Garza-Sánchez et al., 2011).

tmRNA is about 4- or 5-fold larger than tRNA, and the central 4/5, which does not participate in the tRNA-like structure, have a unique pseudoknot-rich secondary structure forming a large ring that surrounds the coding region for tag-peptide (**Figure 1A**; Felden et al., 1997; Nameki et al., 1999b). As several aspects of this novel system became clearer, new questions have arisen. How does tmRNA select the stalled ribosome? How does tmRNA enter the A-site of the ribosome without an anticodon? How does the tag-encoding region of tmRNA substitute for truncated mRNA? How does tmRNA about 5-fold larger than tRNA move in the narrow space of the ribosome to continue translation? How is the resuming point on tmRNA determined? The *trans*-translation reaction can proceed *in vitro* using isolated components from *E. coli* (Shimizu and Ueda, 2002; Ivanova et al., 2004; Konno et al., 2007) or *Thermus thermophilus* (Takada et al., 2007), revealing that a stalled ribosome with a truncated mRNA and a peptidyl-tRNA, Ala-tmRNA, elongation factors and a tmRNA-binding protein called SmpB are the minimal requirement for the first few steps of *trans*-translation including the initial binding of tmRNA to the stalled ribosome, peptidyl-transfer from peptidyl-tRNA to Ala-tmRNA and decoding of the first codon on tmRNA (Kurita et al., 2012). Another tmRNA-binding protein, a ribosomal protein S1 has also been identified (Wower et al., 2000). It is dispensable for *trans*-translation at least until the first peptidyltransfer reaction (Qi et al., 2007; Takada et al., 2007), although it might have a role in a later process of *trans*-translation (Saguy et al., 2007; Shi et al., 2011). S1 is absent in a group of Gram-positive bacteria, although tmRNA still acts in *Bacillus subtilis* belonging to this group (Muto et al., 2000; Ito et al., 2002).

It has recently been revealed that bacterial cells have a variety of systems to rescue the stalled ribosome. Since it is universally conserved in bacteria, *trans*-translation is considered as the primary ribosome rescue system in the bacterial cells. Besides, the *trans*-translation system has distinct features including employment of a small RNA as the main player and addition of a tag for degradation to the nascent polypeptide. In this review, we focused on the molecular mechanism of the apparently spectacular reaction of *trans*-translation involving co-translational mRNA switching.

## SmpB

**FIGURE 2:**
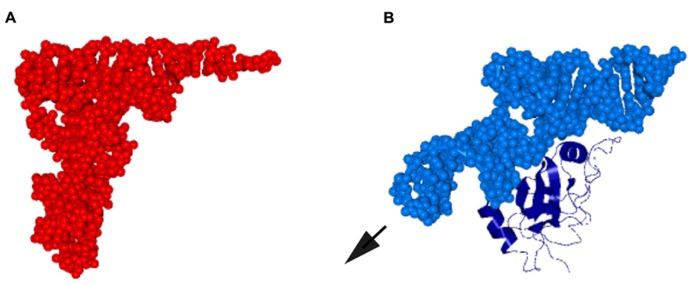
**Molecular mimicry of tRNA by the globular domain of SmpB and TLD. (A)** *Saccharomyces cerevisiae* tRNA^Phe^ represented by space-filling model (PDB ID: 1EHZ). **(B)** A complex of TLD (the 5′ 25 residues and the 3′ 34 residues connected by a UUCG loop) of *T. thermophilus* tmRNA represented by space-filling model and the globular domain (N-terminal 123 of total 144 residues) of SmpB shown in ribbon representation (PDB ID: 2CZJ; Bessho et al., 2007). The remaining region of tmRNA (positions 26–312) is protruded from TLD in a direction similar to that of the long variable arm of class II tRNA, designated by an arrow.

SmpB is essential for* trans*-translation *in vivo* (Karzai et al., 1999) and *in vitro* (Hanawa-Suetsugu et al., 2002; Shimizu and Ueda, 2002). SmpB has a globular core with a C-terminal tail unstructured in solution (Dong et al., 2002; Someya et al., 2003), and the globular domain binds to the tRNA-like domain (TLD) of tmRNA to prevent tmRNA from degradation and enhance aminoacylation of tmRNA (**Figure 1A**; Barends et al., 2001; Hanawa-Suetsugu et al., 2002; Shimizu and Ueda, 2002; Nameki et al., 2005). A crystal structure of a complex of SmpB and a model RNA fragment corresponding to TLD has revealed that the tertiary structure of TLD is indeed similar to the upper half of the L-form structure of the tRNA molecule as has been expected from the tRNA-like primary and secondary structures including the 3′-terminal CCA sequence, the amino acid acceptor stem and T-arm. Intriguingly, the globular domain of SmpB binds to TLD so that it compensates for the lack of the lower half of the L-form structure of tRNA in tmRNA (**Figure 2**; Gutmann et al., 2003; Bessho et al., 2007). When TLD is put on the upper half of the L-form structure of tRNA, the globular domain of SmpB is superimposed on the anticodon arm, indicating that TLD with the globular domain of SmpB structurally mimics a whole tRNA molecule. Functional mimicry of tRNA by TLD·SmpB has been suggested by a directed hydroxyl radical probing study showing that at least two SmpB molecules have capacity to bind an *E. coli *70S ribosome, one to the A-site and the other to the P-site, both of which can be superimposed on the lower half of the tRNA molecules in the elongating ribosome (Kurita et al., 2007). An additional mimicry of the upper half of tRNA by TLD would facilitate full mimicry of each tRNA molecule in the elongating ribosome. SmpB in complex with a TLD fragment facilitates polyalanine synthesis *in vitro* without template mRNA, functionally supporting the molecular mimicry of tRNA and mRNA by TLD·SmpB (Shimizu and Ueda, 2006).

The truncation or mutation of the C-terminal tail of SmpB seriously affects *trans*-translation *in vivo* and *in vitro*, indicating its functional significance (Jacob et al., 2005; Sundermeier et al., 2005; Konno et al., 2007; Kurita et al., 2007, 2010). However, cryo-EM studies have failed to identify the C-terminal tail due to lack of resolution (Gillet et al., 2006; Kaur et al., 2006). Its location in the ribosome has been revealed by a directed hydroxyl radical probing study (Kurita et al., 2007). The C-terminal tail of A-site SmpB extends to the downstream tunnel along the mRNA path. Probing signals from the C-terminal tail of A-site SmpB extends to the downstream tunnel along the mRNA path, and those from its latter half appear at interval of three residues suggesting an α-helical structure. In contrast, probing signals from P-site SmpB are localized in a limited area, and thus it is likely to be in a folded conformation around the mRNA path in the P-site. Thus three different modes of conformations of the C-terminal tail of SmpB have been assumed: an unstructured structure in solution, an extended structure from the A-site to the mRNA entry channel and a folded structure in the P-site (Kurita et al., 2007).

## CURRENT MODEL OF *TRANS*-TRANSLATION

**FIGURE 3:**
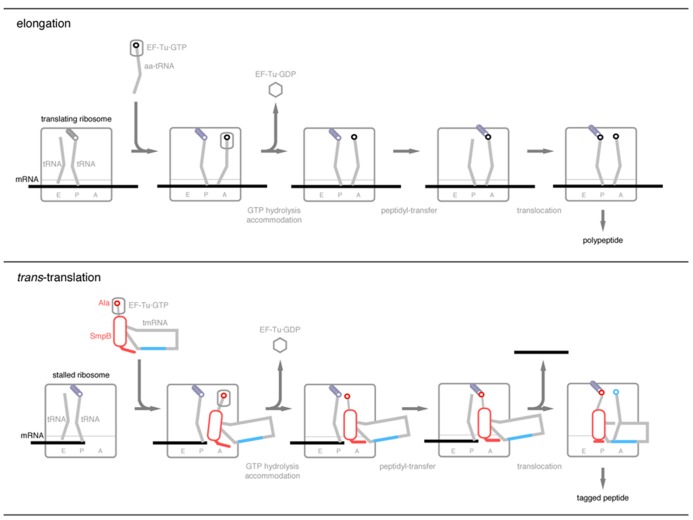
**A current model of *trans*-translation process in comparison with the elongation process of canonical translation.** After GTP hydrolysis by EF-Tu, the C-terminal tail of SmpB becomes located along the mRNA path to recognize the stalled ribosome free of mRNA. Upon translocation of peptidyl-Ala-tmRNA·SmpB from the A-site to the P-site, the C-terminal tail undergoes a drastic conformational change from the extended to the folded structures to accommodate the resume codon of tmRNA into the decoding center. During these processes, TLD and the globular domain of SmpB mimic the upper and lower halves of tRNA, respectively.

Based on the structural resemblance of the complex of SmpB and TLD to a tRNA molecule, the binding sites of SmpB shared by translating tRNA, and the interaction of the C-terminal tail of SmpB with the mRNA path, a model of the *trans*-translation process has been proposed (**Figure 3**; Kurita et al., 2007, 2010). In this model, TLD·SmpB is assumed to functionally mimic the dynamic behavior of tRNA in the elongating ribosome through all the classical and hybrid states, A/T, A/A, A/P, P/P and P/E states.

After aminoacylation with alanine, tmRNA·SmpB binds to EF-Tu·GTP to make a quaternary complex, and thereafter it enters the vacant A-site of the stalled ribosome. Subsequent GTP hydrolysis of EF-Tu may induce conformational changes of the quaternary complex and the ribosome so that EF-Tu·GDP is released and Ala-TLD·SmpB is accommodated in the A-site. During this process, the C-terminal tail of SmpB interacts with the mRNA path downstream from the A-site with an extended structure. This interaction would be successful only when the downstream mRNA is absent. Then Ala-TLD in the A-site receives the nascent polypeptide chain from peptidyl-tRNA in the P-site, and the resulting peptidyl-Ala-TLD·SmpB may translocate from the A-site to the P-site. During this process, the C-terminal tail of SmpB dissociates from the mRNA path and binds to the region around the P-site codon with changing its conformation from the extended structure to the folded structure, which releases mRNA from the ribosome. Just after the movement of peptidyl-Ala-TLD·SmpB to the P-site, the resume codon of tmRNA is positioned at the decoding region.

This model might involve several aspects of the tRNA and mRNA mimicries (1) as the structural unit for entrance to the ribosome with EF-Tu·GTP, (2) as the structural unit for accommodation in the A-site after GTP hydrolysis by EF-Tu, (3) as the structural unit from the A-site to the P-site during translocation, (4) as the mRNA for the first alanine residue of the tag-peptide, and (5) to find the target ribosome in which the downstream mRNA is absent. Although several proteins have been proposed to mimic tRNA such as EF-G (Nissen et al., 1995), SmpB is a special case in that it is assumed to mimic translating tRNA at all the classical and possibly hybrid states from the A-site to the E-site.

## TLD·SmpB IN SOME STATES OF *TRANS*-TRANSLATION COMPLEXES

The above model is consistent with several structural studies. Cryo-EM maps corresponding to four different states (pre-accommodation, accommodation, translocational intermediate, and post-translocated states) during the *trans*-translation process have been revealed, where SmpB and TLD occupy the lower and upper halves, respectively, of the A-site or P-site (Kaur et al., 2006; Cheng et al., 2010; Fu et al., 2010; Weis et al., 2010a,b; Ramrath et al., 2012). In addition to the tRNA mimicry by SmpB and TLD, an interaction of the C-terminal tail of SmpB with the downstream mRNA path from the decoding region has explicitly been shown in a crystal structure of *T. thermophilus* ribosome in complex with a tmRNA fragment, SmpB, EF-Tu·GDP and kirromycin, which mimics the pre-accommodation state of *trans*-translation (Neubauer et al., 2012). Note that kirromycin is effective for some tmRNA fragments such as TLD but much less effective for full-length tmRNA (Shimizu and Ueda, 2006), and thus the kirromycin complex is not likely to perfectly mimic the pre-accommodation state of *trans*-translation. In this crystal structure, the C-terminal tail (residues 122–144) extends to the downstream tunnel along the mRNA path with its latter half (residues 132–144) forming an α-helix as has been suggested by a directed hydroxy radical probing study (Kurita et al., 2007). Some but not all preparations of pre-accommodation state complex contain an additional SmpB molecule near the GTPase associated center of the 50S subunit (Kaur et al., 2006; Weis et al., 2010a), although the relevance of the second SmpB molecule is still controversial (Bugaeva et al., 2008; Felden and Gillet, 2011).

## EARLY STEPS OF *TRANS*-TRANSLATION

**FIGURE 4:**
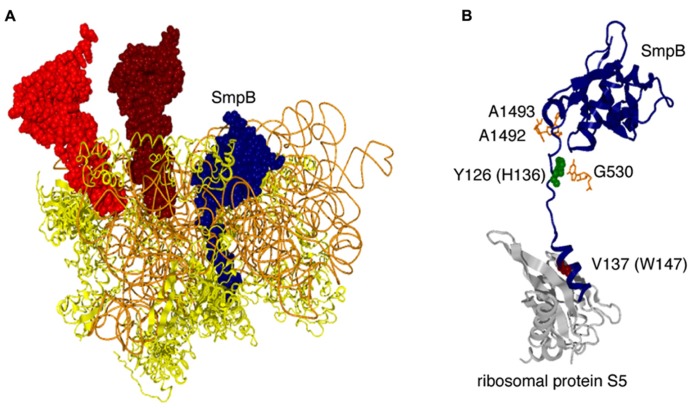
**Location of SmpB in a pre-accommodation state complex of *trans*-translation. (A)** SmpB (dark blue, space-filling model), P-site tRNA (brown, space-filling model), E-site RNA (red, space-filling model), 16S rRNA (light brown, wire model), and the ribosomal proteins (yellow, ribbon model) in a crystal structure of *T. thermophilus* ribosome in complex with a tmRNA fragment, SmpB, EF-Tu·GDP and kirromycin (PDB ID: 4ABR), which mimics the pre-accommodation state of *trans*-translation (Neubauer et al., 2012), are depicted, although the 50S subunit, tmRNA fragment, EF-Tu·GDP and kirromycin are removed. **(B)** SmpB (dark blue, space-filling model), decoding nucleotides (orange, stick model) and the ribosomal protein S5 (gray, ribbon model) are extracted from the same complex. Y126 (green, space-filling model) and V137 (brown, space-filling model) in the C-terminal tail of SmpB are highlighted. The corresponding residues in *E. coli* SmpB are shown in parentheses.

In the elongation process of the canonical translation, a ternary complex (aminoacyl-tRNA·EF-Tu·GTP) enters the elongating ribosome, and only when the anticodon of aminoacyl-tRNA successfully interacts with the codon, GTP is hydrolyzed by EF-Tu to release EF-Tu·GDP from aminoacyl-tRNA and the ribosome. During this process, the codon–anticodon interaction induces conformational change in the decoding center nucleotides, A1492, A1493, and G530 of 16S rRNA, leading to domain closure of the 30S subunit to trigger GTP hydrolysis. However, an alternative interaction should be assumed in the case of *trans*-translation, since it lacks a codon–anticodon interaction. Direct interaction of *E. coli* SmpB with the decoding region has been suggested by chemical modification in combination with NMR (Nonin-Lecomte et al., 2009). In a crystal structure of a *T. thermophilus* pre-accommodation state complex, A1492 and A1493 are in close proximity to the globular domain of SmpB, and G530 stacks with Y126 in the C-terminal tail of SmpB (**Figure 4**), while the ribosomal protein S12, which is deeply involved in decoding fidelity, has no contact with SmpB (Neubauer et al., 2012). Consistently, *in vitro trans-*translation activity is significantly affected by aminoglycosides that flips A1492 and A1493 out of helix 44, while streptomycin, which binds between the juxtaposed residues (C1490 and G1491) and S12, has only a small effect (Takahashi et al., 2003; Konno et al., 2004). Recently, Miller and Buskirk (2014) have found that a mutation in H136 in the C-terminal tail of *E. coli *SmpB causes serious decrease in GTP hydrolysis. The corresponding residue of *T. thermophilus* SmpB, Y126, is involved in stacking with G530 in a crystal structure of a pre-accommodation state complex of *trans*-translation (Neubauer et al., 2012). This may highlight the importance of stacking of H136 with G530 for GTP hydrolysis, although any of A1492, A1493 and G530 in *E. coli *16S rRNA can be changeable without loss of GTP hydrolytic activity as well as peptidyl-transferase activity of *trans*-translation (Miller et al., 2011).

*Trans*-translation is thought to preferentially target the ribosome stalled on a 3′ truncated mRNA, and this situation can be produced by cleavage of intact mRNA around the decoding region. In fact, A-site codon-specific cleavage by RelE (Pedersen et al., 2003) or other unknown endoribonuclease with the help of a 3′ to 5′ exonuclease, RNase II (Garza-Sánchez et al., 2009), has been reported. Besides RelE, several kinds of ribosome-dependent endoribonucleases have been identified in *E. coli *(Feng et al., 2013). The idea that cleavage of mRNA in the stalled ribosome is the prerequisite for *trans*-translation has also been supported by *in vitro* studies: the efficiency of *in vitro* *trans*-translation decreases with the increasing length of the 3′-extension of mRNA from the decoding region (Ivanova et al., 2004; Asano et al., 2005). This can be accounted for by the competition of the 3′-extension of mRNA, if it is longer, with the C-terminal tail of SmpB for the downstream mRNA tunnel. Consistently, the C-terminal tail of *T. thermophilus* SmpB in a crystal structure of a pre-accommodation state complex of *trans*-translation is incompatible with mRNA within a narrow mRNA tunnel (Neubauer et al., 2012). Systematic amino acid substitutions of the C-terminal residues of *E. coli* SmpB have indicated the significance of a tryptophan residue at 147 (W147) in the middle of the C-terminal tail for binding to the mRNA path required for peptidyl-transfer (**Figure 4**; Kurita et al., 2007, 2010). The corresponding residue of *T. thermophilus* SmpB, V137, is involved in hydrophobic interaction with a ribosomal protein S5 in a crystal structure of a pre-accommodation state complex of *trans*-translation (Neubauer et al., 2012). W147 is changeable without significant loss of GTP hydrolysis, suggesting that the interaction of this residue with the mRNA path occurs after GTP hydrolysis (Kurita et al., 2010). Recently, we found that the efficiency of GTP hydrolysis by EF-Tu reflecting that of the entrance of the Ala-tmRNA·SmpB·EF-Tu·GTP complex to the stalled ribosome is comparable regardless of the length of the 3′-extension of mRNA, and that accommodation or rejection of Ala-tmRNA·SmpB from the ribosome stalled on mRNA is determined after GTP hydrolysis depending on the length of the 3′-extension of mRNA (Kurita et al., manuscript in preparation). It is surprising that Ala-tmRNA·SmpB discriminates the target ribosome in a GTP-wasting manner.

EF-Tu·GTP has a role in the proofreading step in which near-cognate aminoacyl-tRNAs that are escaped from the first selection in the initial binding of the ternary complex before GTP hydrolysis are subjected to the second selection after GTP hydrolysis via recognition of the correct codon–anticodon interaction by the decoding region. Unlike tRNAs, tmRNA is homogeneous in a cell, and thus neither codon–anticodon interaction nor proofreading should be required for *trans*-translation. GTP hydrolysis would be required mainly for release of EF-Tu, which guarantees the aminoacylation state of tmRNA.

## FROM THE A-SITE TO THE P-SITE

In the canonical elongation process, peptidyl-tRNA translocates with mRNA from the A-site to the P-site. In *trans*-translation, peptidyl-Ala-tmRNA·SmpB mimicking peptidyl-tRNA, but without mRNA, translocates from the A-site to the P-site. As a consequence, deacylated tRNA and truncated mRNA are pushed from the P-site to the E-site presumably by the globular domain and the folded C-terminal tail of SmpB, respectively, facilitating their dissociations from the ribosome. As in the canonical translation, this process might be driven by EF-G·GTP. In fact, EF-G stimulates release of deacylated tRNA and truncated mRNA (Ivanova et al., 2005). Concomitantly, the resume codon on tmRNA for the tag-peptide should be set at the decoding region. A folded rather than extended conformation of the C-terminal tail of SmpB around the P-site as observed in a directed hydroxyl radical probing study might be preferable for both pushing mRNA out of the P-site and setting the resume codon on tmRNA in the A-site (Kurita et al., 2007). Translocation usually involves the ratchet-like rotation between the two ribosomal subunits (Frank and Agrawal, 2000). Comparison of the cryo-EM structures of the accommodation and post-translocated state complexes have suggested that the ribosome in which peptidyl-Ala-tmRNA·SmpB occupies the A-site also undergoes a ratchet-like rotation upon translocation to induce a significant conformational change of a bridge between the 30S and 50S subunits (bridge B1a), which serves as the barrier between the A-site and P-site, thereby allowing peptidyl-Ala-TLD·SmpB to move from the A-site to the P-site (Weis et al., 2010b; Felden and Gillet, 2011). Consistently, bridge B1a is open in another cryo-EM map of a translocational intermediate complex containing EF-G and fusidic acid (Ramrath et al., 2012). In this complex, latch, which is usually closed by the interaction between the head (helix 34) and body (the G530 region) to form the mRNA tunnel, is open to introduce the coding region of tmRNA into the decoding center (Ramrath et al., 2012). Meanwhile, the large ring comprised of the central four pseudoknot structures (PK1–PK4) of tmRNA (Felden et al., 1997; Nameki et al., 1999b) keeps surrounding the beak of the 30S subunit through the pre-accommodation to the post-translocated states (Fu et al., 2010; Weis et al., 2010b; Ramrath et al., 2012).

## DETERMINATION OF THE RESUME CODON

The coding region for the tag-peptide starts from the position about 10 nucleotides downstream of PK1. Then a question arises as to what determines the first (resume) codon for tag-peptide. It seems reasonable to assume that some sequence or structural unit on tmRNA is fixed somewhere on the ribosome to set the first codon on the decoding center just after translocation. Changing the span between PK1 and the coding region does not affect the resume codon selection, indicating the lack of significance of PK1 for determination of the initiation point (Lee et al., 2001). *In vivo* and *in vitro* studies have revealed the significance of the sequence between PK1 and the tag-encoding region, especially at positions –6 to +1 (Williams et al., 1999; Lee et al., 2001). *In vitro trans*-translation system can identify not only the efficiency of the first peptidyl-transfer of *trans*-translation but also the second amino acid of the tag-peptide, which enables us to specify the start point of the tag-translation (Lee et al., 2001; Konno et al., 2007; Takada et al., 2007; Kurita et al., 2011). Using this system, we have found that some point mutations within the span of -6 to +1 reduce the *trans*-translation efficiency and/or shift the resuming point of translation on tmRNA by -1 or +1, indicating that this region serves not only as the enhancer of *trans*-translation but also as the determinant for the resume codon for the tag-peptide (Lee et al., 2001; Konno et al., 2007).

Then the next question arises as to what recognizes this sequence. It has been shown that U at position -5 is protected from chemical modification by the globular domain of *E. coli* SmpB (Konno et al., 2007). This protection is suppressed by a point mutation in the TLD required for SmpB binding, indicating that a single SmpB molecule bridges two separated domains of tmRNA. The interaction of SmpB with U at -5 would make more sense upon selection of the resume codon for tag-peptide translation in the ribosome. Mutations that induce -1 and +1 shifts of the start point of tag-peptide translation *in vitro* also shift the site of protection at position -5 from chemical modification by -1 and +1, respectively (Konno et al., 2007), indicating the significance of the fixed span between the site of interaction on tmRNA with SmpB and the start point of tag-peptide translation. Thus the interaction between tmRNA and SmpB would be more important for resume point determination than that between tmRNA and the ribosome. A functional interaction of the upstream region in tmRNA with SmpB has been supported by a genetic study showing that a mutation at position -4 of *E. coli* tmRNA that inactivates *trans*-translation is suppressed by some mutations in SmpB (Watts et al., 2009). It is also consistent with current cryo-EM studies suggesting that the nucleotides at -4 and -5 are in close contact with SmpB around the P-site in the post-translocated (resume) state of *trans*-translation (Fu et al., 2010; Weis et al., 2010b). Another *in vivo* study has suggested the importance of the C-terminal tail of SmpB and its interaction with the start GCA codon on tmRNA for determination of the start point of tag-peptide translation (Camenares et al., 2013). This again emphasizes the importance of the interaction between tmRNA and SmpB.

Shift of the start point of tag-peptide translation can also be induced by some kinds of aminoglycosides (Takahashi et al., 2003; Konno et al., 2004), which cause miscoding of translation by binding to A1492 and A1493 to modulate the conformation of the decoding region (A-site).

## DIVERSITY OF RESCUE SYSTEMS OF THE STALLED RIBOSOME

**FIGURE 5:**
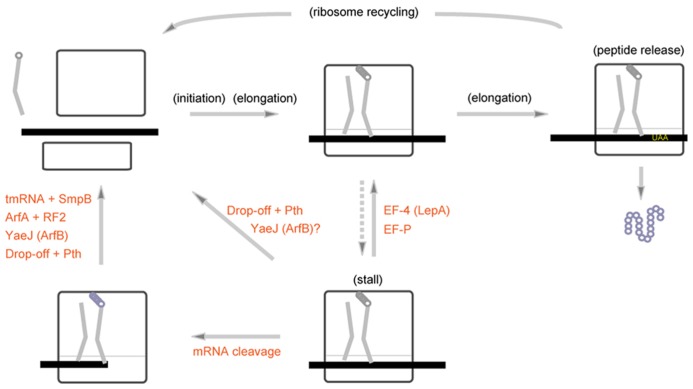
**Multiple pathways to rescue the bacterial stalled ribosome.** Various kinds of mechanisms rescue the stalled ribosome in bacterial cells. Some types of stalled ribosomes are rescued by EF4 (LepA) or EF-P, which facilitates resumption of elongation process. The stalled ribosome with a short nascent peptide can be rescued by drop-off and subsequent peptidyl-tRNA hydrolysis by Pth. ArfA (YhdL) and YaeJ (ArfB) as well as Ala-tmRNA·SmpB would rescue the stalled ribosome probably after cleavage of mRNA. YaeJ (ArfB) would possibly target the stalled ribosome without cleavage of mRNA as well (Shimizu, 2012).

Peptidyl-tRNA often drops-off from the ribosome stalled within the first few rounds of elongation, followed by hydrolysis of detached peptidyl-tRNA by Pth (peptidyl-tRNA hydrolases; Heurgué-Hamard et al., 1998; Gong et al., 2007; Singh et al., 2008). Drop-off of peptidyl-tRNA can also occur at the 3′ end of mRNA (Kuroha et al., 2009) and the temperature-sensitive phenotype of Pth is suppressed by overexpression of tmRNA (Singh and Varshney, 2004). In 2010s, we came to know that the rescue systems of the bacterial stalled ribosome are more diversified than we have imagined (**Figure 5**). For example, EF4 (also known as LepA), which is usually sequestered in the cell membrane, is released into cytoplasm to rescue the translational arrest by back translocation upon high intracellular magnesium ion concentration or low temperature (Pech et al., 2011). The ribosome often stalls at the Pro-Pro-Pro or Gly-Pro-Pro arrest sequence, and EF-P can alleviate this arrest by modulating the peptidyl-transferase center (Doerfel et al., 2013; Ude et al., 2013). Unlike the drop-off/Pth system, EF-4 and EF-P promote resumption of elongation process to complete translation rather than abortively terminate translation. Recently, two stalled ribosome rescue systems, one involving ArfA (also known as YhdL; Chadani et al., 2010) and the other involving YaeJ (also known as ArfB; Chadani et al., 2011b; Handa et al., 2011), have been found. ArfA is a protein that requires RF2 for hydrolysis of peptidyl-tRNA (Chadani et al., 2012; Shimizu, 2012), and YaeJ is a release factor homologue but lacking a stop codon recognition capacity. These findings have encouraged us to approach the whole picture for the bacterial ribosome rescue systems in the cell. Among these ribosome rescue systems, *trans*-translation is the only system that involves a small RNA and is also the only system that is active in preventing truncated mRNA-derived non-functional proteins from accumulation in the cell.

Judging from its absolute occurrence among bacteria, *trans*-translation might be the primary ribosome rescue system in the bacterial cells. Indeed, the ArfA-mediated ribosome rescue system has been suggested to serve as the backup system for *trans*-translation (Chadani et al., 2011a; Garza-Sánchez et al., 2011). Since deprivation of *trans*-translation causes various kinds of disorders of bacterial cells and it is absent in higher eukaryotes, the *trans*-translation process is being used as a target for antibiotic development (Shi et al., 2011; Ramadoss et al., 2013b). A drug that specifically inhibits a step of *trans*-translation would make a contribution to a better understanding of the molecular mechanism of the *trans*-translation process.

## DOWNSTREAM mRNA PATH AS THE PRIMARY DETERMINANT OF THE RIBOSOME RESCUE SYSTEMS

The C-terminal tail of YaeJ is in a situation similar to that of SmpB: it has an unstructured form in solution, although it extends to the downstream mRNA tunnel along the mRNA path with an α-helical structure to hydrolyze the peptidyl-tRNA in the P-site of the stalled ribosome (Gagnon et al., 2012). While the *trans*-translation system is distributed only among bacteria with some exceptions, YaeJ or its homologue (ICT1) is present not only in bacteria but also in mitochondria (Handa et al., 2010; Richter et al., 2010). In eukaryotic cytoplasm, a complex of Dom34p (also known as Pelota) and Hbs1 (Tsuboi et al., 2012), which is structurally similar to the eRF1·eRF3 or aminoacyl-tRNA·EF-Tu complex (Chen et al., 2010), participates in recue of the stalled ribosome. The central and C-terminal domains of Hsb1 have sequence and structural similarities to those of eRF3, while the N-terminal domain is distinct, which appears to recognize the downstream mRNA path of the stalled ribosome (Becker et al., 2011). Thus various types of ribosome rescue machineries would employ the interaction with the vacant downstream mRNA path to recognize the target ribosome.

## Conflict of Interest Statement

The authors declare that the research was conducted in the absence of any commercial or financial relationships that could be construed as a potential conflict of interest.
